# The thrombopoietin mimetic romiplostim leads to the complete rescue of mice exposed to lethal ionizing radiation

**DOI:** 10.1038/s41598-018-29013-5

**Published:** 2018-07-13

**Authors:** Masaru Yamaguchi, Tokuhisa Hirouchi, Koki Yokoyama, Ayaka Nishiyama, Sho Murakami, Ikuo Kashiwakura

**Affiliations:** 1 Department of Radiation Science, Hirosaki University Graduate School of Health Sciences, 66-1 Hon-cho, Hirosaki, Aomori, 036-8564 Japan; 2 Department of Radiobiology, Institute for Environmental Sciences, 2-121 Hacchazawa, Takahoko, Rokkasho-vil. Kamikita-gun, Aomori, 039-3213 Japan

## Abstract

For the primary treatment of emergency exposure to high-dose radiation, such as in the event of a radiation accident, the top priority is the reconstitution and restoration of haematopoiesis. In most radiation accidents, drug therapy is chosen as the most suitable treatment; the chosen drug should already be approved domestically, stably supplied and regularly stockpiled. In the present study, a single administration of romiplostim (RP), an approved thrombopoietin receptor agonist, produced a 100% survival rate in C57BL/6 J mice exposed to a lethal dose (7 Gy) of ^137^Cs γ-rays, and all irradiated mice survived for more than 30 days with both 3- and 5-day consecutive administrations. By day 30, the peripheral blood cells, bone marrow cells and haematopoietic progenitor cells of the RP-administered irradiated mice had all recovered to a level that was not significantly different from that in non-irradiated mice. In contrast to myelosuppression, which did not fully recover until day 30, the expression of several bone marrow cell surface antigens recovered sooner, and DNA repair concurrently increased in haematopoietic cells, speeding the resolution of double strand breaks and reducing the rates of apoptosis. These findings suggest that RP may be a clinic-ready countermeasure to treat victims of radiation accidents.

## Introduction

Severe acute radiation syndrome (ARS) induced by more than 3–4 Gy of radiation exposure can bring about immediate death due to life-threatening multiorgan failure involving the haematopoietic and gastrointestinal systems^1,2^, and in the case of radiation accidents, many victims develop ARS and come close to dying. The priority for ARS is achieving the reconstitution and restoration of haematopoiesis, as radiation-induced bone marrow death frequently results in infections due to a decreased number of white blood cells, bleeding due to a lack of platelets, and anaemia due to a dearth of red blood cells in the circulation^3,4^ within 60 days after irradiation. Furthermore, in cases of high-dose radiation exposure, it is necessary to treat gastrointestinal injury, which can induce nausea, vomiting, loss of appetite and abdominal pain within 1 month after irradiation. Bone marrow transplantation (BMT) and pharmacological approaches are effective and commonly used treatments for haematopoietic failure of ARS^5–8^, but their medicinal applications are clearly different. The use of BMT has many limitations involving a patient’s age, HLA type and post-therapy graft-versus-host rejection, while pharmacological approaches possess relatively few limitations and can be applied to anyone, anytime, anywhere. Thus, pharmacological approaches are ideal countermeasures for radiation emergencies and accidents and would ideally be conducted with commercially available and widely approved pharmaceutical drugs; the best such drug treatment would be a single medication.

In our previous study involving mice lethally irradiated with 7 Gy of γ-rays, we successfully optimized a protocol with multiple approved drugs: romiplostim (RP), erythropoietin (EPO), granulocyte colony-stimulating factor (G-CSF) and nandrolone (ND; 19-nortestosterone)^9^. Although the combination of EPO, G-CSF and ND (no RP) resulted in the survival of only 50% of the irradiated mice at day 30, the survival rate of mice treated with the complete RP-added protocol reached 100%. In addition to granulocyte macrophage colony-stimulating factor and interleukin-3, G-CSF and EPO are known to be potentially effective for accelerating the recovery of patients’ bone marrow, even after exposure to lethal doses of radiation^10–13^. In contrast to G-CSF and EPO, however, the utility of RP as a potential radioprotective agent is unclear^14–16^.

RP is an approved TPO mimetic for the treatment of idiopathic thrombocytopenic purpura (ITP) and promotes the activation of myeloproliferative leukaemia virus proto-oncogene (c-mpl) receptors and the production of platelets^17^. ND, a pharmaceutical drug used in surgery and to treat thermal injuries, has been reported to accelerate the regeneration of the small intestinal mucosa following irradiation^18^. However, the addition of RP greatly improved the survival rate of irradiated mice with a damaged small intestine^9^, suggesting that RP plays a crucial role in the recovery of the haematopoietic and gastrointestinal systems when administered via the optimized protocol.

In the present study, we focused on the efficacy of RP in the rescue of mice exposed to a lethal dose of γ-rays. Because a single administration of RP sufficiently improved the 30-day survival rates of irradiated mice, we further optimized the protocol of single RP administration. During the 30-day post-irradiation period, we evaluated the radiomitigative effects of RP on the haematopoietic and gastrointestinal systems through cellular and molecular analyses.

## Results

### *In vivo* investigation of medication identifies the effectiveness of the thrombopoietin-mimetic c-mpl agonist RP as a countermeasure against ARS

**Figure 1:**
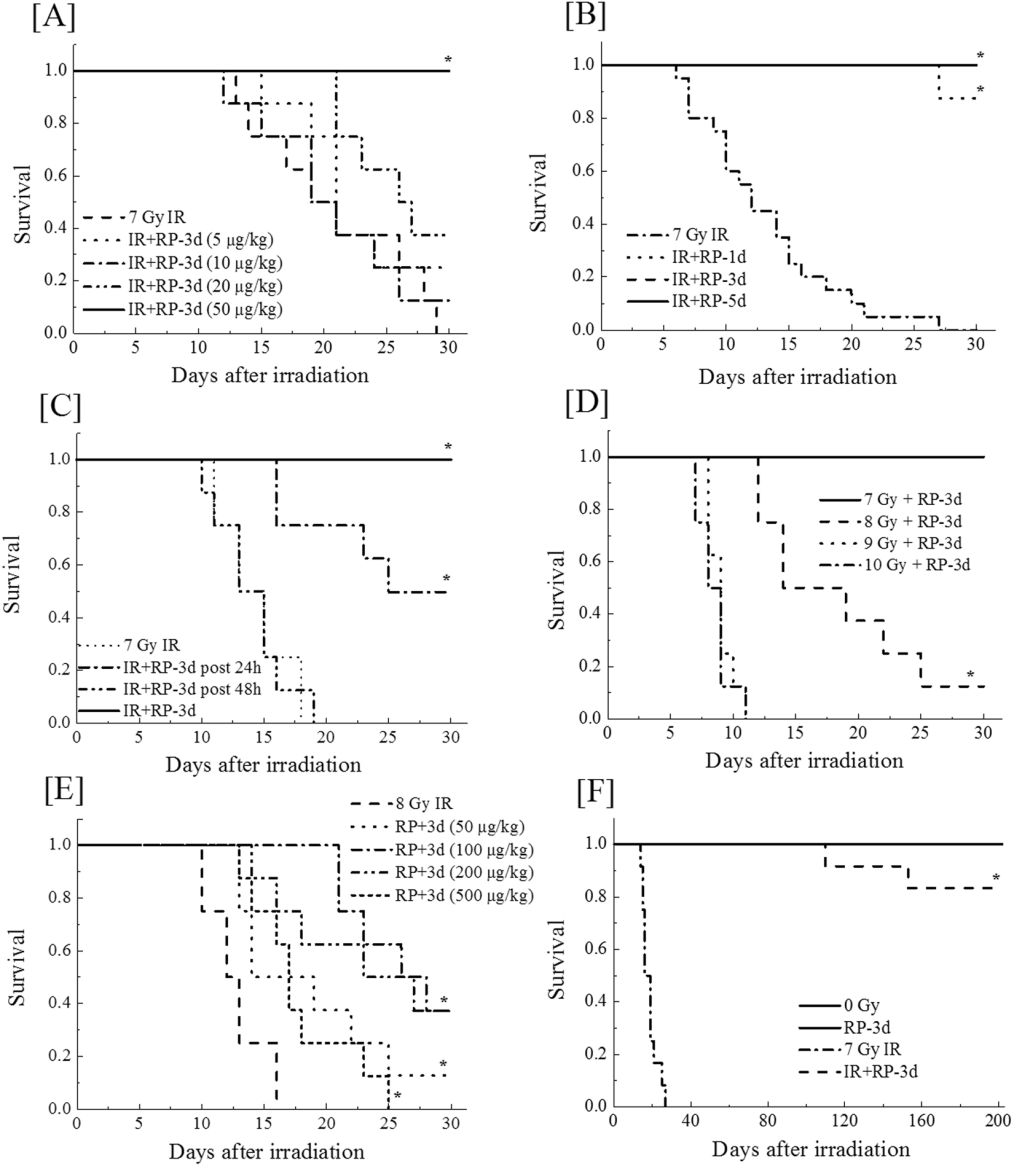
Improvement of post-radiation survival by the TPO-mimetic c-mpl agonist RP. (**A**) Single RP administration dose-dependently mitigated the lethal effect of 7 Gy of γ-irradiation. The dosages of RP were 5, 10, 20 and 50 µg/kg of body weight (µg/kg)/day (“IR + RP” groups), and the administrations were given intraperitoneally for 3 consecutive days, starting immediately after irradiation (within 2 hours). The group receiving no drug treatment is shown as “7 Gy IR,” and the number of mice in each group was eight. Survival data were analysed using Kaplan-Meier survival curves (n = 8 in all the groups). (**B**) Effect of administration length on post-radiation survival. The period of RP administration was varied to 1, 3 and 5 consecutive days (IR + RP-1d, 3d and 5d,n = 12–20 in each group) at identical dosage (50 μg/kg of body weight/day). (**C**) Effect of administration delay. The first of 3 RP administrations on consecutive days was conducted 2, 24 or 48 hours after irradiation (RP, RP post 24 h or 48 h). Each dosage was fixed at 50 µg/kg/day (n = 8 in each group). (**D**) Radiomitigative effects of RP were shown against large doses of γ-irradiation (7 Gy + RP, 8 Gy + RP, 9 Gy + RP, and 10 Gy + RP, n = 8 in all the groups) compared with each radiation control. RP dosages were identical at 50 µg/kg/day. (**E**) Dosage-dependent radiomitigative effect of in 8 Gy-irradiated mice. More than 50 µg/kg/day of RP administration (100, 200 and 500 µg/kg/day) dosage-dependently improved the survival of 8 Gy-irradiated mice (n = 8 in all the groups). (**F**) Long-term observations of RP-treated mice (50 µg/kg for 3 consecutive days). Mice irradiated with 7 Gy and treated with RP (IR + RP-3d) were kept until 200 days after irradiation. “0 Gy”, “RP-3d” and “7 Gy IR” represented results of non-irradiated mice with or without RP administration and non-RP-treated irradiated mice, respectively (n = 16 in each group). Asterisks in panels indicated significant differences by the log-rank test compared with each radiation control (*P* < 0.05).

Our previous study showed that RP was very important for multidrug ARS treatment^9^; thus, the present study investigated the radiomitigative effect of a single administration of RP. Three consecutive days of RP administration mitigated the lethal effect of a 7-Gy dose of γ-irradiation in a dose-dependent manner (Fig. 1A). The radiomitigative effects of RP were similarly shown in mice with 1-day and 5-consecutive-day administration (Fig. 1B), and the drug showed partial effectiveness even if administered 24 hours after irradiation (Fig. 1C). The average post irradiation survival time of mice receiving 24-hour-delayed RP administration was 25 days, significantly longer than that of untreated controls (14 days) but shorter than that of mice that received RP after only a 2-hour delay. These results suggest that the radiomitigative effect of single RP administration depend on administration dosage and duration and are impaired by delay of initial administration; additionally, RP administration on 3 consecutive days, beginning within 2 hours after irradiation, at a dosage of 50 µg/kg of body weight (µg/kg) can almost completely suppress the lethal effect of 7 Gy of radiation.

RP showed slight radiomitigative action against the lethal effect of more than 7 Gy of radiation. The above-mentioned RP regimen suitable for 7 Gy-irradiated mice (3 consecutive days of administration at 50 µg/kg, beginning 2 hours after irradiation) significantly lengthened the average lifetime of 8 Gy-irradiated mice to 19 days, compared to the 12.75 days of 8 Gy-irradiated control mice, and allowed 1 out of 8 irradiated mice to survive for 30 days after irradiation (Fig. 1D). On the other hand, the 7 Gy-suitable RP dose was ineffective against the lethal effects of 9 Gy and 10 Gy. The lifetime-lengthening effect of RP on 8 Gy-irradiated mice was improved by dose optimization (Fig. 1E). Among the administered doses of 50, 100, 200, and 500 µg/kg, the highest 30-day survival rate and longest lifetime were shown in mice that received a dose of 100 µg/kg. The radiomitigative effect of RP against the lethal effects of 8 Gy irradiation seemed to increase in a dose-dependent manner from 50 to 100 µg/kg, but the peaked radiomitigative effect appeared to decrease in a dose-dependent manner above 200 µg/kg.

At the end of a series of investigating the radiomitigative effect of RP, we confirmed the long-term survival of 7 Gy-irradiated mice treated with suitable RP administration. Observation until 200 days after irradiation showed that the 200-day survival of the irradiated mice with RP administration was approximately 85% (Fig. 1F). Side effects of RP were not shown, since all non-irradiated mice that received RP administration survived with no malignancy until 200 days. From these results, we decided in the present study to analyse the radiomitigative effects of RP in 7 Gy-irradiated mice treated with a suitable regimen for that radiation dose.

### Improvement of multiple tissues by RP administration

**Figure 2:**
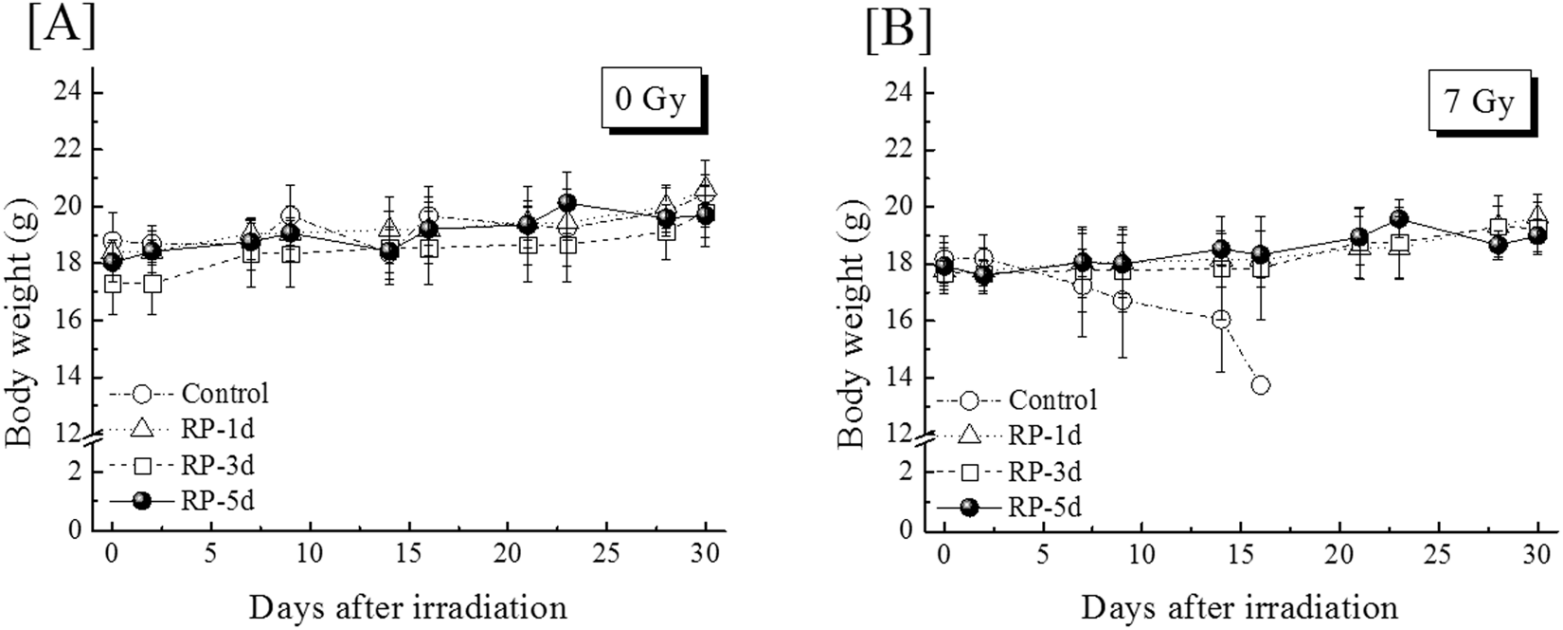
Body weight of surviving mice through day 30. (**A**) and (**B**) show the findings for the non-irradiated and irradiated mice treated with RP for 1, 3 or 5 consecutive days. This experiment was conducted for 30 days. Mice exposed to 7 Gy of radiation or non-irradiated mice without drug treatment were used as controls. The data are expressed as the means ± SD (n = 12).

**Figure 3:**
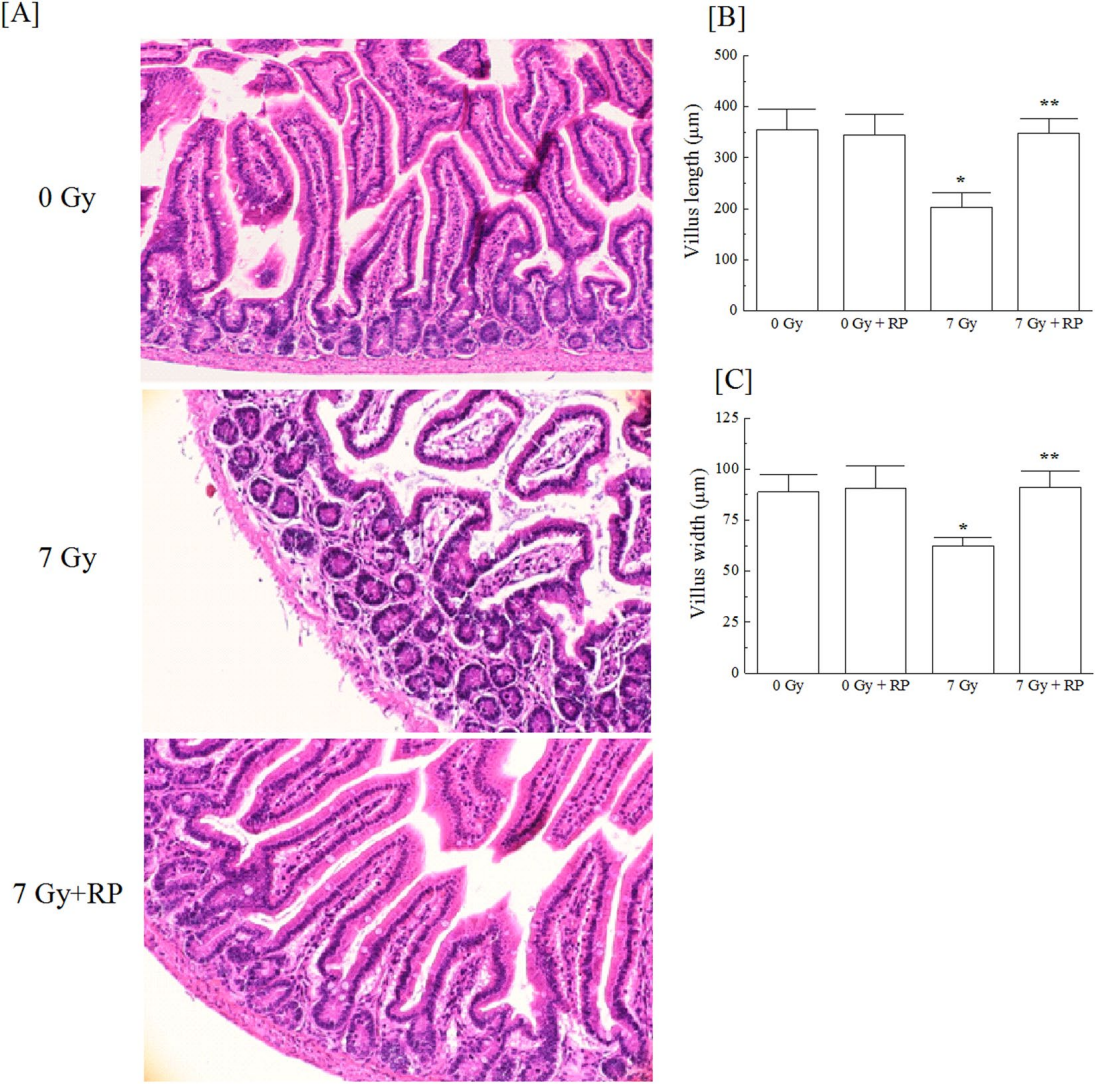
Histology of the small intestine of surviving mice. (**A**) Histological sections of the small intestine from non-irradiated control, RP-untreated irradiated and RP-treated irradiated mice on day 10 were stained with haematoxylin and eosin stain (20× magnifications). (**B**) The villus length and (**C**) villus width were measured. The data are expressed as the means ± SD, and statistical significance was determined by a comparison of each group (*P < 0.05 vs. 0-Gy cohort; **P < 0.05 vs. 7-Gy cohort).

The body weights of the non-RP-treated irradiated mice gradually decreased across 17 days after irradiation (Fig. 2B). By contrast, the administration of RP for 1, 3 or 5 consecutive days of RP administration suppressed the body weight reduction caused by irradiation (Fig. 2). The effect of RP on the small intestine is shown at day 10 (Fig. 3A–C). Figure 3A shows representative small intestines of non-irradiated (RP-untreated) and irradiated mice without and with RP treatment at day 10. Regarding mice without RP treatment, the villi remained showing radiation-induced denudations and morphological alterations. However, the small intestines of the RP-treated mice regained their morphological integrity. While the villus length of the RP-untreated irradiated mice was significantly reduced by 43% of the value from non-irradiated controls, the villi of the RP-treated mice similar in length to those of non-irradiated controls (Fig. 3B). Likewise, the villi of the RP-treated irradiated mice were similar in width to those of the non-irradiated controls (Fig. 3C). These findings indicate that RP promoted the recovery of gastrointestinal tissues damaged by γ-irradiation.

**Figure 4:**
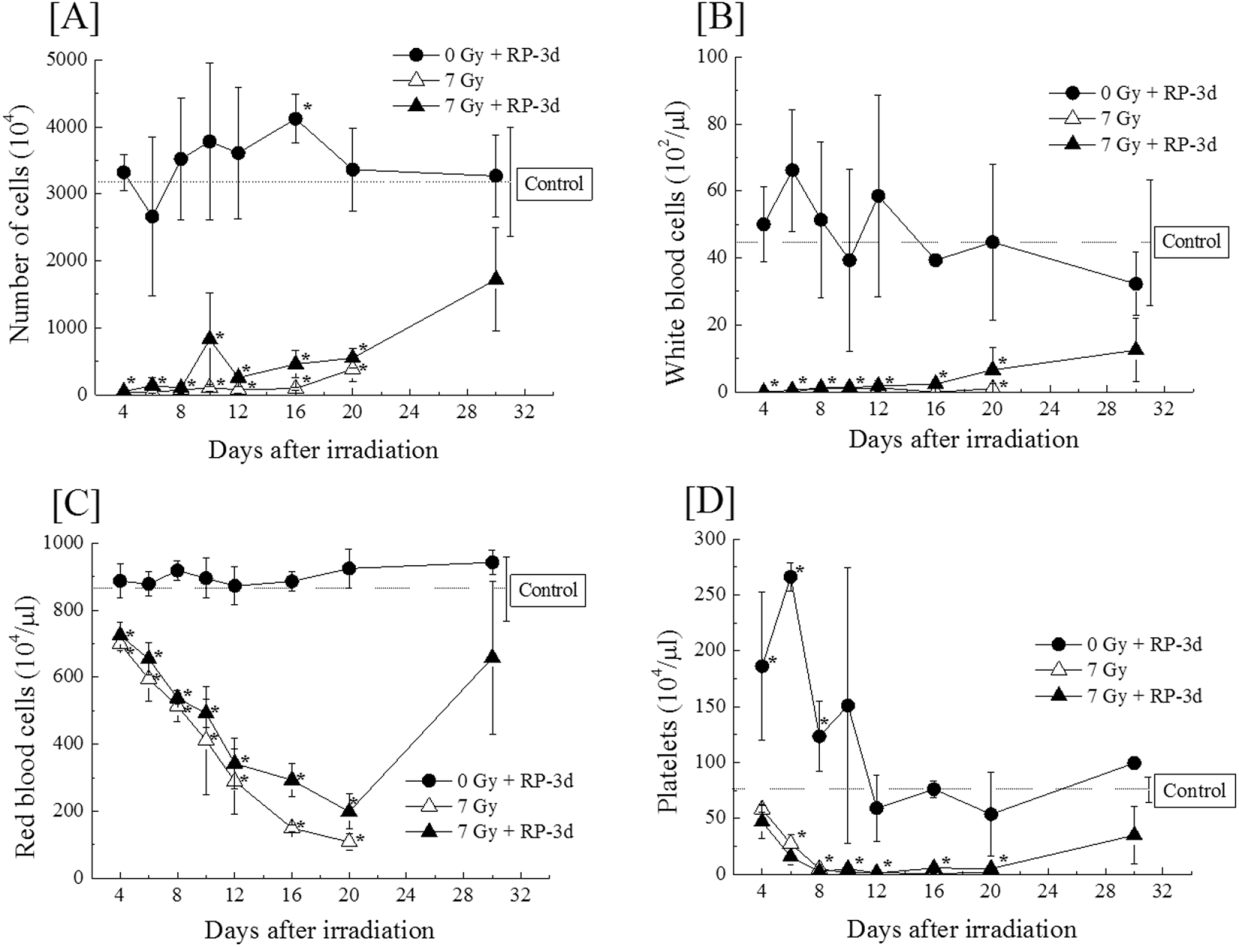
The mean numbers of bone marrow cells in the femur and mature haematopoietic cells in the peripheral blood. (**A**) The effects of RP treatment for 3 consecutive days on the bone marrow of 7-Gy irradiated mice were estimated by comparing the total numbers of nucleated bone marrow cells in the treated mice and the non-irradiated control mice after irradiation. The total numbers of nucleated bone marrow cells in the femurs were counted using Burker-Turk solution. The dotted line in the figure shows the value for non-RP-treated, non-irradiated mice. The data are expressed as the means ± SD (n = 12–30). Panels (**B–D**) show the mean numbers of white blood cells, red blood cells and platelets, respectively. The dotted line in each figure shows the value of non-RP-treated, non-irradiated mice. The data are expressed as the means ± SD (n = 12–30). Statistical significance was determined by a comparison of each irradiated group with the non-irradiated control mice (*P < 0.05).

The haematopoietic system was also restored with RP treatment. Independently of RP treatment, bone marrow cell counts were significantly decreased within 4 days after irradiation and remained impoverished until day 20 (Fig. 4A). Recovery from the decreased number of bone marrow cells occurred from day 20 to 30 with RP treatment, and bone marrow cells in irradiated mice with RP treatment reached counts that were statistically similar to those in non-irradiated mice. Regarding peripheral blood, the white blood cell, red blood cell, and platelet counts of the irradiated mice were all decreased by irradiation and recovered with RP treatment (Fig. 4B–D). However, there was a difference in the response to irradiation among peripheral blood cells: white blood cells behaved similarly to bone marrow cells (Fig. 4B) but decreases in red blood cell and platelet counts gradually progressed until at least day 20 (Fig. 4C,D). Interestingly, RP treatment markedly enhanced platelet production, but platelets recovered at approximately the same time as white and red blood cells and bone marrow cells. At day 30, the number of peripheral blood cells (white and red blood cells and platelets) was incompletely recovered in the irradiated mice, but there were no significant differences between control and RP-treated irradiated mice (Table 1).

**Table 1:** The total numbers of bone marrow cells in the femurs of surviving mice, mature haematopoietic cells in the peripheral blood, and haematopoietic progenitor cells at day 30.

	Dose	Control	RP-1d	RP-3d	RP-5d
Bone marrow cells(×10^4^ cells)	0 Gy	3327 ± 815	3465 ± 235	3268 ± 611	4790 ± 947
	7 Gy	—	1381 ± 876	1720 ± 773	2380 ± 1007
White blood cells (×10^2^ cells/μL)	0 Gy	60.6 ± 30.0	34.0 ± 6.16	32.2 ± 9.46	29.0 ± 15.0
	7 Gy	—	15.7 ± 11.9	12.5 ± 9.46	9.31 ± 5.58
Red blood cells (×10^4^ cells/μL)	0 Gy	841 ± 223	848 ± 117	942 ± 37.0	964 ± 55.2
	7 Gy	—	612 ± 163	658 ± 227	740 ± 93.8
Platelets (×10^4^ cells/μL)	0 Gy	84.2 ± 24.6	90.2 ± 26.2	99.6 ± 5.53	110 ± 8.66
	7 Gy	—	37.8 ± 25.9	35.0 ± 25.9	28.8 ± 17.1
Haematopoietic progenitors (×10^4^ cells)	0 Gy	11.9 ± 2.95	19.4 ± 8.71	19.1 ± 10.4	14.6 ± 3.84
	7 Gy	—	2.11 ± 2.04	3.58 ± 4.41	2.96 ± 3.17

**Figure 5:**
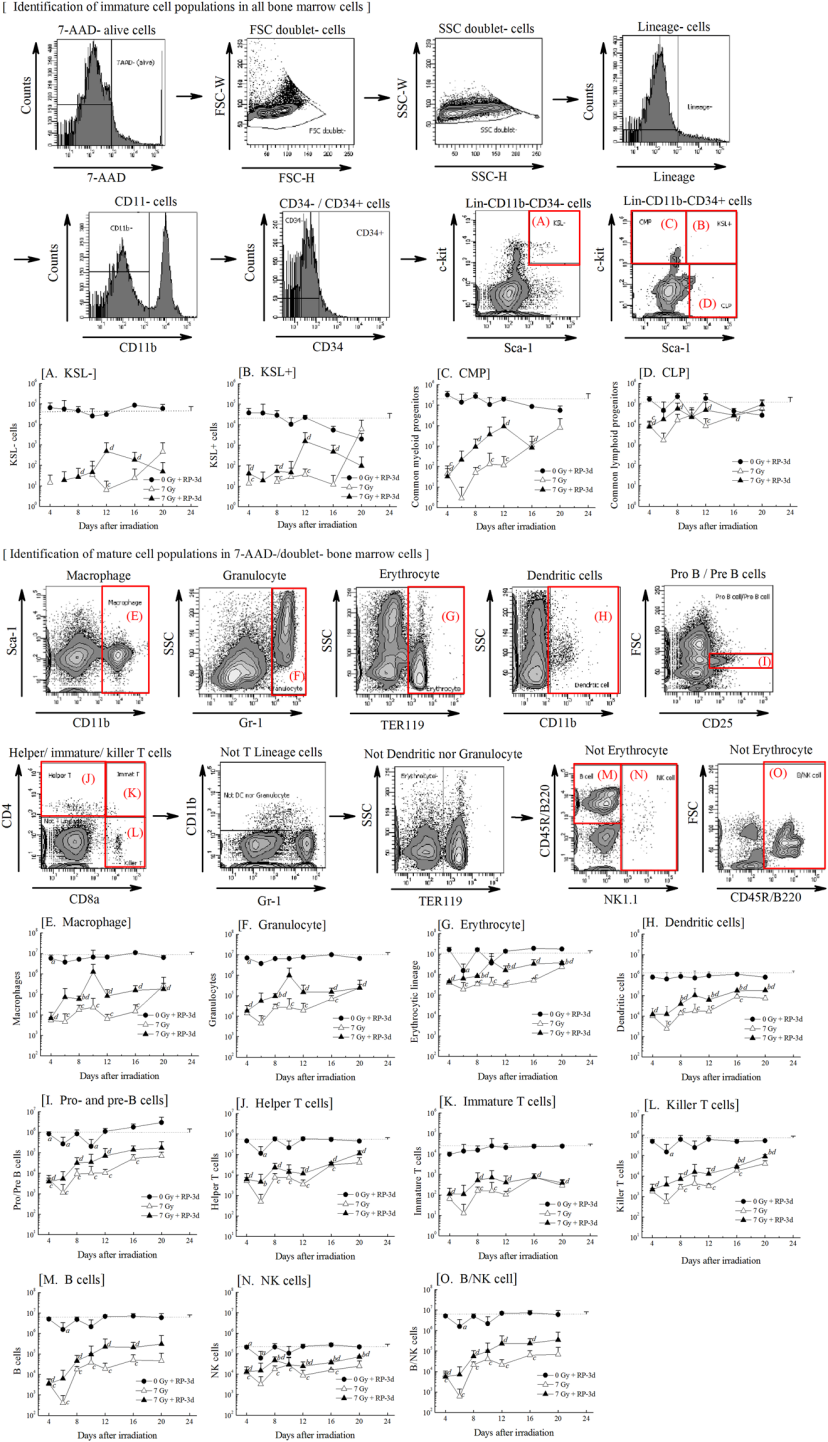
The cell numbers of 15 populations in the bone marrow of surviving mice. The nucleated bone marrow cells in the femurs of the non-irradiated and irradiated groups with or without RP were classified into 15 populations according to their cell surface antigen profiles. The representative FACS histograms and plots of the non-irradiated control at day 0 showed how to identify the immature cell populations (**A**–**D**) and mature cell populations (**E**–**O**). The doublet-discriminated fraction of the 7-AAD^−^ populations were used for the analysis. Populations negative for lineage markers and CD11b were confined to the population enriched for haematopoietic stem and progenitor cells (Lin^−^c-kit^+^Sca-1^+^CD34^−^: KSL^−^) (**A**), multipotent progenitors (Lin^−^c-kit^+^Sca-1^+^CD34^+^: KSL^+^) (**B**), common myeloid progenitors (Lin^−^c-kit^+^Sca-1^−^CD34^+^: CMP) (**C**), and common lymphoid progenitors (Lin^−^c-kit^−^Sca-1^+^CD34^+^: CLP) (**D**). On the other hand, in mature cells, the doublet-discriminated fractions of the 7-AAD^−^ populations were used for the identification of macrophages (CD11b^+^) (**E**), granulocytes (Gr-1^+^) (**F**), erythroid progenitors (TER119^+^) (**G**), dendritic cells (**H**), pro- and pre-B cells (CD25^+^) (**I**), helper T cells (CD4^+^CD8a^−^) (**J**), immature T cells (CD4^+^CD8a^+^) (**K**), and killer T cells (CD4^−^CD8a^+^) (**L**). B cells (CD45R/B220^+^NK1.1^−^) (**M**), NK cells (NK1.1^+^) (**N**) and B/NK cells (**O**) were isolated from non-T lineage cells. The dotted line in each figure shows the value for RP-untreated non-irradiated mice. The data are expressed as the means ± SD (n = 12–30 used in each group). Statistical significance was determined by a comparison among (a) the non-irradiated groups with and without RP, (b) the irradiated groups with and without RP, (c) the non-irradiated and irradiated groups, and (d) the RP-treated groups with and without irradiation (*P* < 0.05).

Moreover, we analysed the haematopoietic system in bone marrow. Haematopoietic progenitor cell counts (total number of CFU-GM, CFU-Mix and BFU-E) in the bone marrow of irradiated mice were insufficiently restored by RP treatment, as were mononuclear cells, whose numbers remained almost identical (Table 1). The nucleated bone marrow cells in the femurs of the non-irradiated and irradiated groups with and without RP were classified into 15 populations according to the cell surface antigen profiles. The representative FACS histograms and plots of the non-irradiated control at day 0 show how to identify the immature cell populations (Fig. 5A–D) and mature cell populations (Fig. 5E–O). The numbers of the population enriched for haematopoietic stem and progenitor cells (Lin^−^c-kit^+^Sca-1^+^CD34^−^), haematopoietic multipotent progenitor cells (Lin^−^c-kit^+^Sca-1^+^CD34^+^) and common myeloid progenitors (Lin^−^c-kit^+^Sca-1^−^CD34^+^) in the bone marrow were significantly lower in RP-untreated irradiated mice than in RP-untreated non-irradiated mice until day 20 (Fig. 5A–C). RP administration did not reverse the radiation-induced reductions in the population enriched for haematopoietic stem and progenitor cells, haematopoietic multipotent progenitor cells, and common myeloid progenitors to restore the levels seen in in RP-untreated irradiated mice. Regarding common lymphoid progenitors (Lin^−^c-kit^−^Sca-1^+^CD34^+^), similar levels were found between RP-untreated irradiated and RP-treated irradiated mice, and there were no significant differences on day 20 (Fig. 5D). The findings for myeloid and lymphoid cells are shown in Fig. 5E–O. The mature myeloid and lymphoid cells were relatively recovered, in contrast to the immature haematopoietic cells. The numbers of macrophages, granulocytes, erythroid progenitors and dendritic cells in the bone marrow were significantly recovered in RP-treated irradiated mice compared to RP-untreated irradiated mice until day 20 after γ-irradiation (Fig. 5E–H). Although the numbers of B cells, immature T cells and helper T cells did not recover following treatment (Fig. 5I–K,M,O), significant recovery was observed for NK cells and killer T cells on a comparison between RP-treated irradiated and RP-untreated irradiated mice (Fig. 5L,N).

### RP administration improves DNA repair response and reduces apoptotic signals

**Figure 6:**
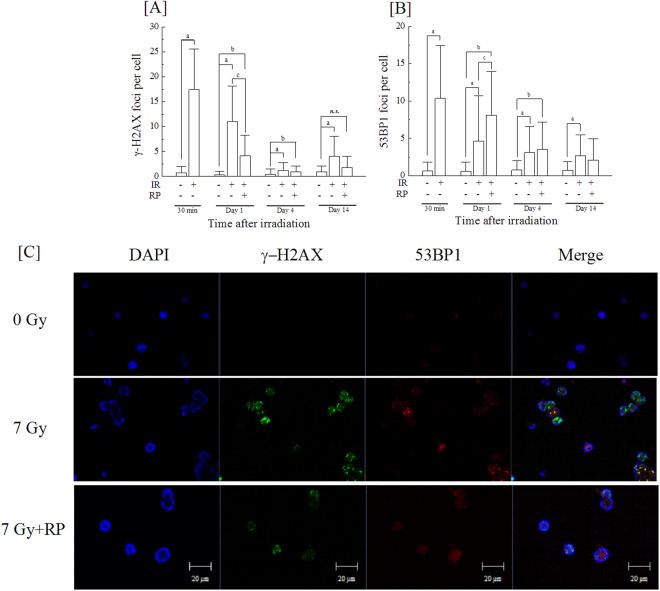
DNA double-strand breakage and DNA repair response in nuclei of bone marrow cells from the femurs of surviving mice. The nucleated bone marrow cells in the femurs of non-irradiated and irradiated groups with or without RP were collected at days 0, 1, 4 and 14, and the expression of (**A**) γ-H2AX and (**B**) 53BP1 was evaluated. (**C**) A representative image from day 1 is shown. The data are expressed as the means ± SD (n = 3 independent experiments). Statistical significance was determined by a comparison between (a) the non-irradiated and irradiated groups without RP, (b) the non-irradiated and irradiated groups with RP, and (c) the irradiated groups without and with RP (*P* < 0.05); “*n.s*.” means “not significant”.

**Figure 7:**
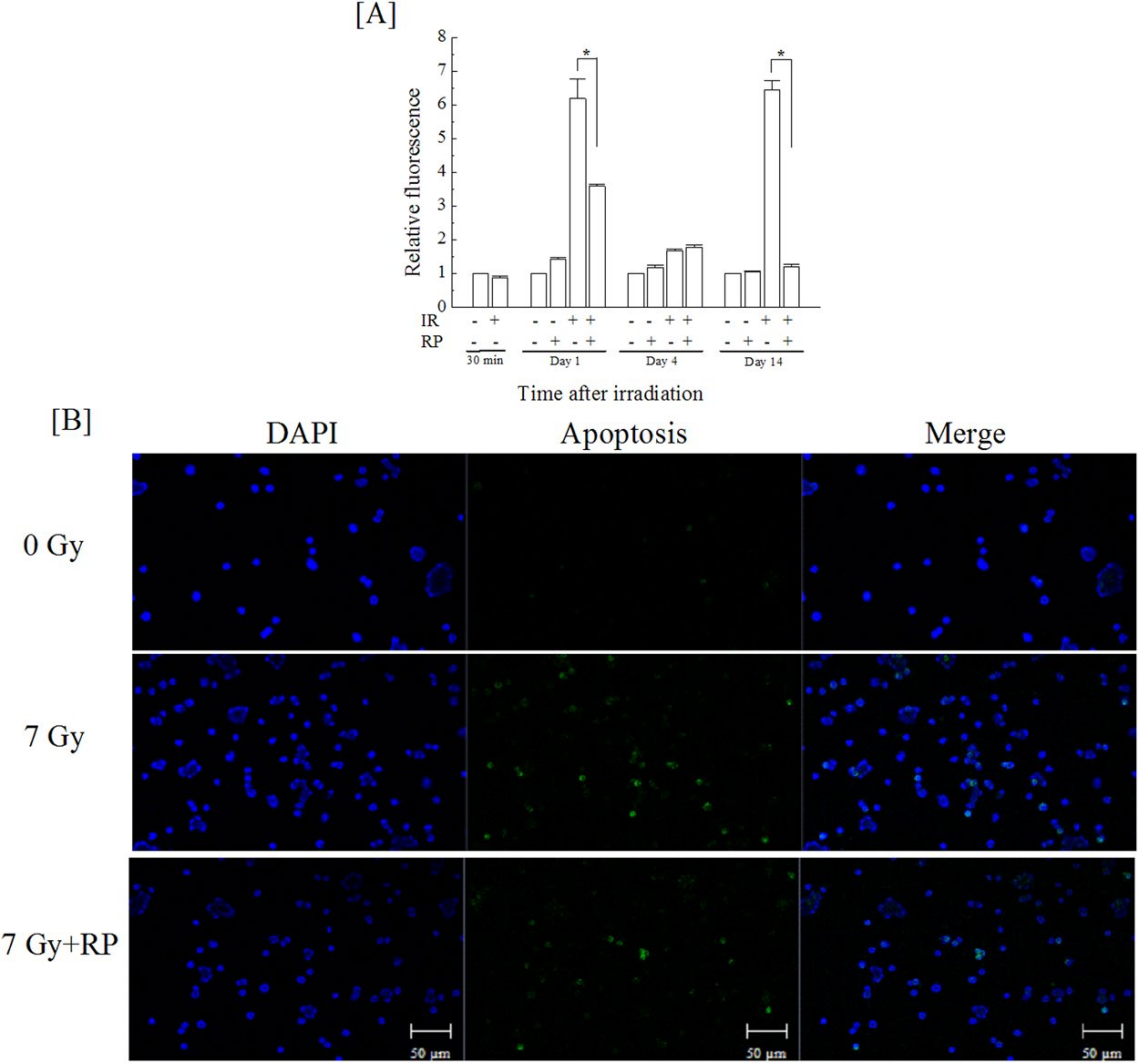
Apoptotic response in the bone marrow cells from the femurs of surviving mice. (**A**) Nucleated bone marrow cells from the femurs of non-irradiated and irradiated groups with or without RP were collected at days 0, 1, 4 and 14, and the cells were tested for apoptosis. (**B**) A representative image from day 1 was shown. The data are expressed as the means ± SD (n = 3 independent experiments). Statistical significance was determined between RP-untreated and RP-treated irradiated mice (*P < 0.05).

To evaluate the effect of RP administration on repair of DNA damage, we analysed the foci of γ-H2AX, one of DNA double strand break markers^19,20^, and 53BP1, one of the non-homologous end joining (NHEJ) factors^21–23^, in bone marrow mononuclear cells of irradiated mice at days 0, 1, 4 and 14, and a representative image for day 1 is shown (Figure 6). γ-H2AX foci were increased 1 day after irradiation and gradually decreased until day 14, but RP administration significantly suppressed the number of γ-H2AX foci at day 1 (Fig. 6A,C). By contrast, radiation-induced 53BP1 foci appeared at day 1 but were significantly increased by RP treatment (Fig. 6B,C). The increase in 53BP1 foci indicated the possibility that the NHEJ pathway was promoted by RP; thus, we analysed the rate of early apoptotic cells among bone marrow mononuclear cells by detecting cytokeratin 18 foci (Fig. 7). Specific antibodies against cytokeratin 18 is used for determination of early apoptotic events in cells and/or tissues by detection of specific cytokeratin 18 fragments containing the aspartic acid residue 396 neo-epitope, which is exposed after cleavage of cytokeratin 18 by caspases. Cleavage at this position is carried by caspase-9 early in apoptosis and by caspase-3 and caspase-7 during the execution phase^24,25^. Cytokeratin 18+/early apoptotic cells populations were significantly increased by irradiation on days 1 and 14 (Fig. 7A,B). Radiogenic apoptosis was significantly suppressed by RP treatment. These results showed that RP administration promoted a DNA repair pathway and suppressed apoptosis.

## Discussion

The present study demonstrated that RP, an ITP therapeutic drug, achieved the complete rescue of mice exposed to lethal γ-irradiation (Fig. 1). A series of analyses of the haematopoietic parameters indicated that RP enhanced the recoveries of several haematopoietic cells by day 30 post-irradiation (Fig. 2,4,5). In addition, RP treatment for 3 days after irradiation improved intestinal integrity and morphology, indicating the restoration of the intestinal mucosa (Fig. 3). Furthermore, at one day after irradiation, DNA double-strand breakage was found to be significantly suppressed after RP treatment, and a significant increase in DNA repair was seen in the nuclei of bone marrow cells, suggesting that RP treatment reduced the apoptosis response of haematopoietic cells nearly to control values (Fig. 6, 7). In the present study, it was shown that the optimum dose of RP was 50 μg/kg of body weight/day (Fig. 1A), which is 50-fold higher than the clinically used dose (1 μg/kg of body weight/day) but within the range showing no toxicity^26^. Moreover, in our preliminary trials using X-rays to overcome mortality due to 8 Gy of irradiation, mice that received 50 µg/kg of RP twice per day (12-hour intervals) for 3 consecutive days achieved a 100% 30-day survival rate (data not shown). Therefore, the present findings suggest that RP may be a very useful radiomitigative drug for the emergency treatment of victims exposed to high doses of radiation and may be especially effective in case of radiation accidents.

The TPO receptor c-mpl is constantly expressed on the surface of haematopoietic stem/progenitor cells^27^ and not only promotes megakaryocytic differentiation but also regulates their self-renewal activity and pluripotency^28,29^. This receptor is classified as a cytokine receptor class I and induces receptor dimerization and tyrosine phosphorylation, as well as a series of signalling events, including the activation of Janus kinase (JAK)-signal transducer and activator of transcription, Shc/Ras/mitogen-activated protein kinase and phosphatidylinositol-3 kinase/Akt^15,30^, which plays a critical role in the signalling of a wide array of cytokines and growth factors leading to various cellular functions, including cell survival, proliferation, growth, haematopoiesis and the immune response^31,32^. De Lavel *et al*. reported that TPO-increased DNA-dependent protein kinase-dependent DNA repair limits haematopoietic stem and progenitor cell mutagenesis in response to DNA damage^33^, and TPO was recently shown to promote NHEJ DNA repair in haematopoietic stem cells through the specific activation of extracellular signal-regulated kinase (Erk) and nuclear factor-κB (NF-kB) pathways and their target, LEX-1^34,35^. In addition, thrombopoietin receptor agonists, such as TPO and eltrombopag, protect against cell death/apoptosis in cardiac myocytes via the down-regulation of caspase-3 activity, which is downstream of JAK/Src tyrosine kinase^36^, and Tronik-Le *et al*. reported the first demonstration of efficacy of a single injection of TPO shortly after *in vivo* exposure to ionizing radiation for reducing HSC injury and improving functional outcomes^37^. TPO has been shown to exhibit important selective DNA repair promoting activity for NHEJ but not homologous recombination. In addition, a few previous reports have shown that TPO and TPO receptor agonists act as radioprotective/mitigative agents^14–16^, and RP was recently approved as a second-generation thrombopoietin agonist^38^. In the present study, RP treatment significantly suppressed DNA double-strand breakage and increased DNA repair in bone marrow cells (Fig. 6), and RP suppressed the accumulation of cytokeratin 18 fragments via cleavage by caspase (Fig. 7), indicating that the RP signal might attenuate the radiation-induced DNA damage, restore the genomic integrity of haematopoietic cells, and suppress the rate of cellular apoptosis. These mechanisms may have resulted in a significant improvement in the 30-day survival rate and the recovery of several haematopoietic parameters (Figs 1–5). However, in order to clarify the details of the mechanisms underlying the increased survival, further studies are needed, such as determining the roles of other important haematopoietic organs, the spleen^39^, and the lung^40^; the roles of secondary internal signalling mechanisms, such as extracellular vesicles^41^; and the therapeutic effects of mesenchymal stromal/stem cells^42^ induced by RP administration, as the survival of an individual exposed to lethal ionizing radiation depends not only on the cell populations and DNA repair in bone marrow cells but also on various factors in various other organs. Additional investigations are currently underway to clarify how the RP-treated mice were able to escape radiation-induced early death immediately after irradiation.

The present study shows that RP leads to the complete rescue of mice exposed to lethal ionizing radiation, with improvement in several haematopoietic parameters and the histological appearance of the intestine. In addition, RP treatment attenuated the radiation-induced DNA damage in nuclei of haematopoietic cells and improved DNA repair, which reduced the rate of apoptotic haematopoietic cells, suggesting that RP may act as a triggered physiological regulator in exposed individuals. These findings suggest that RP may be useful as a clinic-ready countermeasure for radiation accidents.

## Materials and Methods

### Exposure of mice to a lethal dose of γ-irradiation

Female C57BL/6 J Jcl mice were delivered at 7 weeks of age from the breeding facilities of Clea Japan (Tokyo, Japan). At 8 weeks of age, the mice were subjected to varying lethal total-body irradiation doses of 7–10 Gy of ^137^Cs γ-rays at a dose rate of 0.9 Gy/min or 7 Gy of X-rays (150 kVp, 20 mA, 0.5 mm aluminium and 0.3 mm copper filters) at a dose rate of 1.0 Gy/min using an X-ray generator (MBR-1520R; Hitachi Medical Co., Tokyo, Japan). The mice were then administered the medications described below. All of the mice were housed in standard cages in a temperature-controlled room under a 12-h/12-h light/dark cycle in a specific-pathogen-free environment at the Institute for Environmental Sciences. They were provided with sterilized standard laboratory mouse chow diet and drinking water *at libitum*. All experiments were conducted according to legal regulations in place in Japan and the Guidelines for Animal Experiments of the Institute for Environmental Sciences and Hirosaki University after approval by the animal experimental committees of both organizations. In the present study, select criteria were applied prior to sacrifice, e.g., more than 20% loss of body weight, respiratory distress, etc.

### Treatment with the human thrombopoietin-mimetic c-mpl agonist RP

The irradiated mice were administered the human thrombopoietin-mimetic c-mpl agonist RP (Romiplate®; Kyowa Hakko Kirin, Co., Ltd., Tokyo, Japan) within 2 h after γ-irradiation. Similarly, the non-irradiated mice were also administered RP and used as controls. RP was administered intraperitoneally, and the doses were determined based on the findings of previous reports^9,13,15,16^. The applied dose of RP used in the present study ranged from 5 to 500 µg/kg/day for 1, 3 or 5 days within 2 h following exposure to γ-irradiation. The treated mice were maintained until day 30 and weighed every week. Until day 30, the numbers of white blood cells, red blood cells and platelets in the peripheral blood of the surviving mice were counted using Celltac-α (NIHON KOHDEN, Tokyo, Japan). The surviving mice were anaesthetized with isoflurane, a widely used inhalation anaesthetic (Powerful Isoful®; Zoetis, London, UK), and sacrificed. Total nucleated bone marrow cells in the femurs of the non-irradiated and irradiated groups with or without RP were counted using Burker-Turk solution (Nacalai Tesque, Kyoto, Japan).

### Profiling the cell differentiation of bone marrow cells using fluorescence-activated cell sorting (FACS)

Cell differentiation profiles of bone marrow cells were analysed using FACSAria (Becton Dickinson, Franklin Lakes, NJ, USA). The bone marrow cells harvested from the mice were treated with Pharm Lyse buffer (Becton Dickinson). After removal of the lysed red cells, 2.5 × 10^5^ cells of the bone marrow cell suspension were stained with fluorescence-labelled CD11b, CD34, Sca1, c-kit, CD25 and a lineage cocktail involving biotin conjugated-CD8a, CD45R/B220, CD4, Ly6G (Gr-1), TER119, CD11c and NK1.1. Next, 2.5 × 10^5^ cells obtained from the identical suspension were stained with fluorescence-labelled CD8a, CD45R/B220, CD4, Ly6G (Gr-1), TER119, CD11c and NK1.1. Following staining with 7AAD, we gated the 7AAD^–^viable cell population and counted the number of Lin^–^c-kit^+^Sca-1^+^CD34^–^ (population enriched for haematopoietic stem and progenitor cells), Lin^–^c-kit^+^Sca-1^+^CD34^+^ (multipotent progenitor), Lin^–^c-kit^+^Sca-1^–^CD34^+^ (common myeloid progenitor), Lin^–^c-kit^–^Sca-1^+^CD34^+^ (common lymphoid progenitor), CD11b^+^ (macrophages), Gr-1^+^ (granulocytes), TER119^+^ (erythroid progenitor), CD4^+^CD8a^+^ (immature T cells), CD4^+^CD8a^–^ (helper T cells), CD4^–^CD8a^+^ (killer T cells), CD25^+^ (pro- and pre-B cells), CD45R/B220^+^NK1.1^–^ (B cells) and NK1.1^+^ (NK cells) populations. Fluorescein isothiocyanate (FITC)-conjugated anti-mouse Sca-1 monoclonal antibodies (mAbs), allophycocyanin-cianin-7-forochrome (APC-Cy7)-conjugated anti-mouse CD11b mAbs, Texas Red-conjugated anti-mouse CD45/B220 mAbs, phycoerythrin-cyanin-7-forochrome (PE-Cy7)-conjugated anti-mouse CD4 mAbs, APC-Cy7-conjugated anti-mouse CD8a mAbs, phycoerythrin (PE)-conjugated anti-mouse CD8b mAbs, phycoerythrin (PE)-conjugated anti-mouse CD25 mAbs, FITC-conjugated anti-mouse Gr-1 mAbs, allophycocyanin (APC)-conjugated anti-mouse TER119 mAbs, biotin-conjugated anti-mouse TER119 mAbs, PE-Texas Red-conjugated streptavidin and 7-AAD were purchased from Becton Dickinson. APC-conjugated anti-mouse c-Kit mAbs, biotin-conjugated anti-mouse CD11b mAbs, biotin-conjugated anti-mouse Gr-1 mAbs, biotin-conjugated anti-mouse CD45R/B220 mAbs, biotin-conjugated anti-mouse CD4 mAbs, biotin-conjugated anti-mouse CD8a mAbs, and biotin-conjugated anti-mouse CD8b mAbs were purchased from BioLegend (San Diego, CA, USA). PE-Cy7-conjugated anti-mouse CD34 was purchased from Santa Cruz Biotechnology (Dallas, TX, USA).

### Methylcellulose culture

Colony-forming cells (CFCs), including colony-forming unit-granulocyte/macrophage (CFU-GM), burst-forming unit-erythroid (BFU-E) and colony-forming unit-granulocyte/erythroid/macrophage/megakaryocyte (CFU-Mix) cells, were assayed by the methylcellulose method as described previously^9^.

### Histological analyses

At day 10 after γ-irradiation or RP treatment, the duodenum tissue was excised from the small intestine at 4 cm below the pyloric junction, flushed with calcium- and magnesium-free phosphate-buffered saline (PBS [−]; Sigma-Aldrich, Stockholm, Sweden), and fixed in 10% formalin overnight. Sections of duodenum tissues (approximately 1 cm in thickness) were embedded in paraffin. Then, 4 μm-thick sections were stained with haematoxylin and eosin (H&E) and observed under 20× magnification using an inverted microscope. The villus height was determined by measuring the distance from the villus-crypt junction to the villus tip, and the villus width was determined by the distance of the villus-crypt junction. Under blinded conditions, the villus length and width were measured for at least 10 villi in every sample.

### Immunofluorescent detection of γ-H2AX, 53BP1

The bone marrow cells were collected at days 0, 1, 4, and 14, washed with PBS (−), and fixed with ice-cold 75% ethanol for 10 min at room temperature. Fixed cells were washed with PBS (−), permeabilized in 0.5% Triton X-100 (Wako, Osaka, Japan) on ice for 5 min, and washed twice with PBS (−). The cells were then incubated with an anti-phospho-histone H2AX (ser139) monoclonal antibody and anti-53BP1 monoclonal antibody (Millipore, Billerica, MA, USA) diluted 1:300-fold with 20 mM Tris-HCl [pH 7.4], 137 mM NaCl, 0.1% TWEEN-20 (TBST) containing 5% skim milk at 37 °C for 120 min, and subsequently washed with PBS (−) and incubated with Alexa Fluor 488 goat anti-mouse IgG (H + L) antibody and Alexa Fluor 546 goat anti-mouse IgM (μ chain) antibody diluted 1:400-fold with TBST containing 5% skim milk at 37 °C for 60 min. Following a second wash with PBS (−), the cells were adhered to microscope glass slides (Matsunami Glass Ind., Osaka, Japan) using a StatSpin® CytoFuge 2 (Iris Sample Processing, Inc., Westwood, MA, USA) and mounted using Vectashield® Mounting Medium with DAPI (Vector Laboratories, Inc., Burlingame, CA, USA). For the quantitative analysis, the γ-H2AX and 53BP1 foci were counted per cell using an LSM 710 laser scanning microscope (Carl Zeiss Microscopy Co., Ltd., Tokyo, Japan) with a Z-stack function that scans by changing the depth for thick samples and storing the data as layers. Under blinded conditions, the number of γ-H2AX and 53BP1 foci per cell was counted for more than 50 cells in every sample.

### Detection of apoptosis

The extent of apoptosis was determined by M30 CytoDEATH antibody (Roche Life Science, Pleasanton, CA, USA) and goat anti-mouse IgG antibody (H + L) FITC conjugate (Millipore) according to manufacturer’s instructions. The bone marrow cells were collected at days 0, 1, 4 and 14; washed with PBS (−); and fixed with ice-cold pure methanol for 30 min at −20 °C. Fixed cells were washed with washing buffer (PBS [−] containing 0.1% TWEEN-20) twice and then incubated with M30 CytoDEATH antibody diluted 1:50 with incubation buffer (PBS [−] containing 1% bovine serum albumin and 0.1% TWEENs-20) for 60 min at 20 °C. These cells were subsequently washed with PBS (−) twice and incubated with anti-mouse IgG-FITC antibody diluted 100-fold with incubation buffer for 30 min at 20 °C. Following a second wash with PBS (−), the cells were adhered to microscope glass slides using a StatSpin® CytoFuge 2 and mounted using Vectashield® Mounting Medium with DAPI. Photographs of the cells were taken with an LSM 710 laser scanning microscope. In addition, a quantitative analysis was performed using a flow cytometer (Cytomics FC500; Beckman-Coulter, Fullerton, CA, USA).

### Statistical analyses

The statistical significance of differences among multiple groups was assessed using the Steel-Dwass test. Data from survival studies data were analysed using the Kaplan-Meier method followed by the Mantel-Cox (log-rank) test for the assessment of significant differences. The levels of significance were calculated using the software program Excel 2007 (Microsoft, Redmond, WA, USA) with the Statcel 3 add-on (OMS, Saitama, Japan). A *P* value of less than 0.05 was taken to indicate statistical significance.
